# Developing Efficient and Accurate Polarizable Molecular Simulation Models for Studying Refrigerants and Their Binary Mixtures

**DOI:** 10.1021/acs.jpcb.6c03275

**Published:** 2026-07-22

**Authors:** Haihui Wang, Ying-Lung Steve Tse

**Affiliations:** Department of Chemistry, 26451 The Chinese University of Hong Kong, Sha Tin, New Territories, Hong Kong, China

## Abstract

Hydrofluoroolefins (HFOs) are promising low-global-warming-potential refrigerants, but the scarcity of mixture data limits reliable prediction of their phase behavior and thermodynamic properties under practical operating conditions. Here, we develop a data-efficient polarizable molecular modeling strategy for HFO-1234yf, HFO-1234ze­(E), and their binary mixtures by combining high-level quantum-mechanical potential-energy data with a limited set of condensed-phase experimental properties, including liquid density, heat capacity, and enthalpy of vaporization. The resulting force fields reproduce key thermodynamic properties of the pure fluids, including isobaric heat capacities and enthalpies of vaporization, over broad temperature ranges, with most deviations within 5% of experimental values. The models also accurately describe vapor–liquid equilibrium behavior across wide temperature and composition ranges, predicting mixture saturation pressures with a mean absolute error of 0.43 bar and surface tensions of the pure fluids and equimolar blend with a mean absolute error of 1.36 mN/m. Structural, energetic, and free-energy analyses reveal that fluorinated-group nonbonded interactions govern volatility and nonideal mixing, leading to preferential enrichment of HFO-1234yf in the vapor phase. Overall, this work demonstrates that polarizable force fields refined using high-level quantum data and minimal experimental input can provide accurate, transferable predictions for refrigerant blends in data-sparse regimes, offering a practical framework for molecular design and screening of next-generation low-GWP refrigerants.

## Graphical abstract

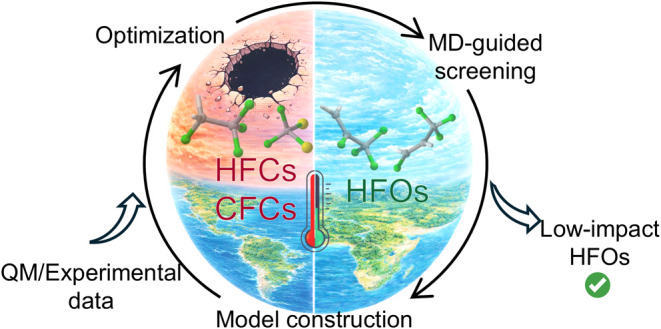

## Introduction

The rising global demand for cooling is expected to significantly increase energy use and greenhouse gas emissions. According to a joint report by the International Energy Agency (IEA) and the United Nations Environment Programme (UNEP), without effective policy intervention, the use of cooling-related energy could triple by 2050,^1^ potentially contributing up to 0.4 °C of global warming^1,2^ due to hydrofluorocarbon (HFC) emissions and fossil fuel-based electricity generation. To mitigate this impact, a transition to fourth-generation refrigerants, hydrofluoroolefins (HFOs), has been proposed. These compounds contain a carbon–carbon double bond, which significantly reduces their atmospheric lifetimes and, consequently, results in a low global warming potential (GWP).^3^ Therefore, they are considered promising alternatives for small-scale commercial and residential cooling systems. Among them, HFO-1234yf (2,3,3,3-tetrafluoropropene) and HFO-1234ze­(E) (trans-1,3,3,3-tetrafluoropropene) are two of the most prominent compounds, which are widely adopted by manufacturers, such as Honeywell, for use in both automotive and stationary air conditioning (AC) systems.^4^ Due to the high performance of refrigerant blends, the formulation such as R-459B, a ternary mixture consisting of 21% HFC-32, 69% HFO-1234yf, and 10% HFO-1234ze­(E), has been proposed as potential replacements for HFC-410A because it offers similar cooling capacity with a lower GWP.^5^ Hence, a comprehensive understanding of their thermophysical properties would facilitate the design of high-performance refrigerant blends. In particular, vapor–liquid equilibrium properties, including saturated liquid and vapor densities, vapor pressure, critical properties, and enthalpy of vaporization, also referred to as latent heat, are essential for evaluating refrigeration-cycle performance, compressor sizing, and heat-transfer behavior. Interfacial properties such as surface tension are also important because they affect phase-change heat transfer, bubble nucleation, and flow distribution in evaporators and condensers. However, the thermophysical behavior of HFO-based mixtures remains under investigation, with the aim of better guiding their use in next-generation cooling technologies.

While HFOs show great potential, their development is hindered by gaps in thermophysical data. Experimentally, substantial data are available for pure refrigerants. However, a recent survey by Bell has pointed out that data on refrigerant mixtures remain very limited.^6^ This data scarcity makes it more challenging to generalize or extend experimental findings to other compositions of mixtures or operating conditions. Fortunately, molecular dynamics (MD) simulation is a powerful tool for generating thermophysical data while providing molecular-level insight into refrigerant behavior.

Raabe^7,8^ developed a transferable force field for fluoropropenes by optimizing force field parameters using the method proposed by Sum et al.,^9^ which involves perturbing the geometry of molecular substructures and comparing the resulting force-field energies with quantum-mechanical (QM) reference data. This force field has been employed to investigate vapor–liquid equilibrium (VLE) properties of various pure and blended refrigerants through Gibbs Ensemble Monte Carlo (GEMC) simulations.^10–14^ Another important modeling method is the polar Perturbed Chain Statistical Associating Fluid Theory (PC-SAFT),^15^ which has been utilized by Vega et al. to predict the thermophysical and interfacial properties of pure third- and fourth-generation refrigerants.^16^ Building on these, Fouad demonstrated that transport properties are strongly influenced by molecular size, structure, and long-range interactions.^17^ In parallel, polar PC-SAFT models have been parametrized to describe HFO systems with improved accuracy; these include predictions of VLE and interfacial properties for both pure substances and mixtures.^18^ In addition to these theoretical approaches, the REFPROP software developed by NIST is widely regarded as the industry standard for refrigerant property calculations and is often used as a benchmark for validating new simulation models and numerical methods.^19^

Although various methods have been developed to study the properties of refrigerant blends, many still face limitations. For example, SAFT models require reference data,^20,21^ either from experiments or MD simulations, to fine-tune parameters for different refrigerants. At the same time, existing models are sufficiently accurate for engineering design, but their parameters are often fitted to only one or two data sets, typically over a limited temperature or composition range.^6^ Conversely, MD offers theoretical flexibility across broader conditions, but its accuracy can be hindered by classical force fields that may not be able to perform well in both the gas and liquid phases, leading to poor predictions of VLE and other interfacial properties.^22^ To address phase coexistence, GEMC simulation avoids explicit interfaces by simulating separate liquid and vapor boxes, allowing particle swaps and volume changes governed through Markov-chain Monte Carlo moves.^23^ While GEMC is effective in capturing isolated phase behaviors, its computational efficiency is limited due to the inherently low acceptance rate of particle swaps and poor parallel scalability. Moreover, interfacial properties, such as surface tension, cannot be measured directly because there are no explicit interfaces.

A unified modeling approach that balances accuracy, efficiency, and generalizability remains elusive. A primary reason to study refrigerants using MD with an explicit interface rather than GEMC is efficiency. MD simulations can be more readily parallelized, carried out on graphics processing units (GPUs), and do not involve rejected moves. Although various force fields have been developed for HFO refrigerants used in GEMC calculations, many were parametrized and validated without an explicit interface. This does not mean such force fields are incompatible with molecular simulations involving an explicit interface; rather, further validation of their suitability for such interface simulations would be advisible. In addition, it is now known that explicit electronic polarizability is important for accurately describing interfacial properties. Therefore, a polarizable force field specifically parametrized and validated for VLE simulations with an explicit interface for the HFO refrigerants studied is desired. To address these concerns, we propose a multiscale strategy to develop a polarizable classical force field, with parameters optimized against high-level reference data from density functional theory (DFT) calculations at the ωB97M-V/def2-QZVPP level.^24,25^ Nonetheless, even though models trained exclusively on DFT-derived gas-phase data may yield highly accurate energy predictions in the gas phase, there can be deviations in certain liquid properties. Therefore, a small number of readily available experimental data in the liquid phase (e.g., liquid density, heat capacity, and heat of vaporization) are incorporated into the optimization process.

## Computational Details

### Polarizable Force Fields

The functional form of our polarizable force fields is based on the general AMBER force field (GAFF)^26^

1 $$U_{\mathrm{sys}}=\sum_{\mathrm{bonds}}k_{b}{\left(r-r_{0}\right)}^{2}+\sum_{\mathrm{angles}}k_{a}{\left(\theta -\theta_{0}\right)}^{2}+\sum_{\mathrm{dihedrals}}k_{d}\left[1+\cos\left(n\phi -\gamma \right)\right]+\sum_{i<j}k_{e}\frac{q_{i}q_{j}}{r_{\mathrm{ij}}}+\sum_{i<j}4\epsilon_{\mathrm{ij}}\left[{\left(\frac{\sigma_{\mathrm{ij}}}{r_{\mathrm{ij}}}\right)}^{12}-{\left(\frac{\sigma_{\mathrm{ij}}}{r_{\mathrm{ij}}}\right)}^{6}\right]+\sum_{\mathrm{Drudes}}k_{D}d^{2}$$

where the force constants *k*_b_ and *k*_a_, the equilibrium bond length *r*_0_, the equilibrium bond angle θ_0_, the Lennard–Jones (LJ) parameters σ_*ij*_ and ε_*ij*_, and the partial charge *q*_*i*_ are adjustable in our strategy. *k*_e_ is the Coulomb constant and *k*_d_ is the torsional force constant. *n* and γ are the parameters used to tune the periods and phases of torsional angles. The Drude oscillator model^27^ was employed to account for the polarization effect in the simulation. This introduces an additional term that calculates the harmonic bond energy between the Drude particle and its parent atom, with force constant *k*_D_ = 1000 kcal·mol^–1^·Å^–2^ and displacement *d*. In constant NVT dynamics, each Drude particle had a mass of 0.4 amu, taken from its parent atom.

### Loss Function

Our force fields were optimized by minimizing the loss function *L*, consisting of contributions from potential energy (PE) differences, density ρ, isobaric heat capacity *C*_P_, and heat of vaporization Δ*H*

2 $$L=w_{1}\left\langle \delta {\mathrm{PE}}^{2}\right\rangle +w_{2}\delta_{\rho }^{2}+w_{3}\delta_{C_{P}}^{2}+w_{4}\delta_{\Delta H}^{2}$$

where the first term on the right is

3 $$\left\langle \delta {\mathrm{PE}}^{2}\right\rangle =\sum_{i=1}^{N_{\mathrm{frame}}}{\left(\Delta {\mathrm{PE}}_{i}-\left\langle \Delta \mathrm{PE}\right\rangle \right)}^{2}/N_{\mathrm{frame}}$$

4 $$\Delta {\mathrm{PE}}_{i}={\mathrm{PE}}_{i,m}-{\mathrm{PE}}_{i,\mathrm{ref}}$$

with subscripts “m” and “ref” referring to the model and the DFT reference, respectively. As a compromise between computational efficiency and preserving the representativeness of the system’s physical behavior, a gas-phase cluster of 10 molecules was formed for each pure system, and an equimolar mixture of 5 molecules each was formed for the binary system. In addition, MD simulations were performed for both pure and binary systems to select 50 configurations with the lowest potential energies in the GFN2-xTB model using Molclus 1.12 in combination with xtb 6.7.1,^28–30^ and these configurations were further optimized at the B3LYP/def2-SVP level with Grimme’s D3­(BJ) dispersion correction using Gaussian 16, Revision C.02.^25,31–34^ Finally, the potential energy values of these optimized reference configurations were calculated more accurately using the ωB97M-V functional with the def2-QZVPP basis set using ORCA 6.0.0.^24,25,35^

The rest of the terms on the right in eq 2 are the scaled deviations from the experimental reference values

5 $$\delta_{X}^{2}={\left(\frac{X_{m}-X_{\mathrm{expt}}}{X_{\mathrm{expt}}}\right)}^{2}$$

where *X* = ρ, *C*_P_, or Δ*H*. The subscripts “m” and “expt” refer to the model prediction and experimental value, respectively. The model values were obtained from constant NPT simulations, and the experimental values were obtained at 300 K and the corresponding saturation pressure.^36–43^ For the binary system, due to the lack of available data under saturated conditions, experimental values^44^ at 300 K and 20 bar were used instead, and the Δ*H* term was omitted. For pure systems, the weights *w*_1_, *w*_2_, *w*_3_ and *w*_4_ were empirically determined through preliminary grid searches and set to 15 (kcal/mol)^−2^, 5000, 5000, and 5000, respectively. For the binary systems, *w*_1_, *w*_2_, and *w*_3_ remained the same, and *w*_4_ was set to 0 because experimental Δ*H* data for the binary mixtures are not readily available. Our training used data at a single temperature (300 K) to demonstrate that our models can make reasonable predictions when extrapolated across a temperature range, which is especially important when experimental data are scarce.

### Optimization

With the loss function in eq 2 defined, the following reparameterization procedure was carried out. The MD simulations in the procedure were all carried out using OpenMM 8.1.0.^45,46^ Two simulation systems with periodic boundary conditions (PBCs) containing 100 molecules were constructed using PACKMOL,^47^ for HFO-1234yf and HFO-1234ze­(E), respectively, based on their experimental densities. The systems were then equilibrated for at least 500 ps under constant NPT (constant number of molecules, pressure, and temperature) dynamics at 300 K and their saturation pressure, which is 5.24 bar for HFO-1234ze­(E) and 7.21 bar for HFO-1234yf.^36,39^ The LJ potential was smoothly switched to zero from *L*/2 – 0.1 nm to *L*/2, where *L* is the lateral box length (i.e., *L* = *L*_*x*_ = *L*_*y*_). The long-range Coulomb interactions were treated using the Particle Mesh Ewald method^48–50^ with a real-space cutoff of *L*/2 and a fractional error tolerance of 10^–5^ in the forces. The extended Lagrangian integration scheme was applied to enable efficient simulation of Drude particles using dual Langevin thermostats^51,52^ to keep the core atoms at the system temperature with a friction coefficient of 10/ps and the Drude particles at 1 K with a friction coefficient of 20/ps. Additionally, the pressure was maintained using the Monte Carlo barostat in OpenMM. Following equilibration, at least 500 ps of production data were collected for optimization. The time step for all the simulations for optimization was 1 fs.

The liquid density ρ was calculated by

6 $$\rho =\frac{M_{\mathrm{molar}}N}{N_{A}V_{\mathrm{NPT}}}$$

where *M*_molar_ is the molar mass of the target molecule, *N* is the number of molecules in the simulation system, *N*_A_ is Avogadro’s constant, and *V*_NPT_ is the average volume of the system in the constant NPT simulation.

Junmei’s method^53^ was applied to calculate the heat of vaporization Δ*H*

7 $$\Delta H=E_{g}^{\min}-E_{l}+\left(\frac{3N_{\mathrm{atom}}-6-N_{\mathrm{con}}}{2}\right)\mathrm{RT}$$

where *E*_g_^min^ is the potential energy of an isolated single molecule after energy minimization, *N*_atom_ is the number of atoms in the system. *N*_con_ is the number of system constraints, and $E_{l}$ is the average potential energy per molecule in the liquid phase.

The heat capacity was then calculated based on the fluctuations in *H* ^54^

8 $$C_{P}=\frac{\sigma^{2}\left(H_{\mathrm{NPT}}\right)}{k_{B}T^{2}}$$

where σ^2^(*H*_NPT_) is the variance of the calculated enthalpy in the constant NPT simulation, *k*_B_ is the Boltzmann constant, and *T* is the system temperature.

Following our previous work,^55^ the loss function was minimized using the Covariance Matrix Adaptation Evolution Strategy (CMA-ES),^56^ a stochastic, derivative-free optimization method based on evolutionary principles. The initial force field parameters for the pure systems were generated using the general automated atomic model parametrization (GAAMP) method,^57^ which is based on QM calculations of isolated molecules in the gas phase. The binary system was optimized only after the two individual HFOs had been optimized. In the binary system, the parameters for the individual HFOs remained the same, but new LJ interaction parameters between the two HFOs were initialized by the Lorentz–Berthelot mixing rules and then optimized.

Upon convergence of the loss function (Figure 1), the five models with the lowest scores were selected for comparison over a range of temperatures. The model that showed the best agreement with the experimental data was chosen for further simulations.

**Figure 1:**
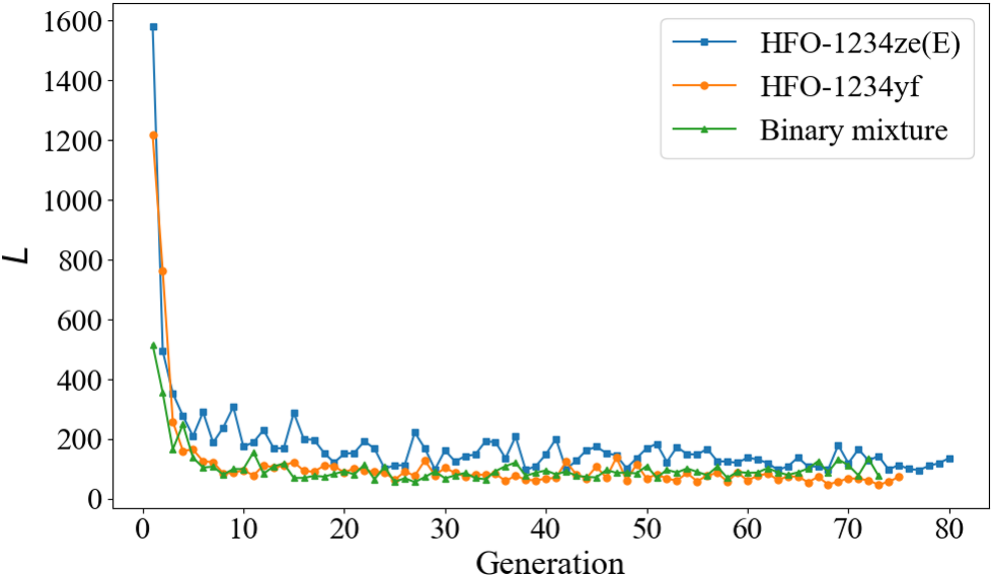
Loss function *L* is plotted as a function of the generation number for the different HFO systems. The binary mixture corresponds to the equimolar HFO-1234yf/HFO-1234ze­(E) mixture.

### MD Simulations with Optimized Models

A liquid slab in contact with its vapor phase was set up to calculate the VLE properties by MD simulations. For a pure system, 500 molecules were randomly placed within a periodic cubic box by PACKMOL,^47^ with the simulation box volume chosen to match the experimental liquid density at 300 K. Then, the length along the *z*-axis of the box was tripled, creating the vapor phase with the liquid slab.

Each MD simulation with the optimized model was equilibrated for at least 40 ns using a 0.5 fs time step in constant NVT dynamics. Five equilibrated configurations from the last 20 ns were selected as the initial states for five subsequent production runs, each lasting another 20 ns. The binary systems were constructed in the same manner as the pure systems, but with a total of 1000 molecules. The mole fraction of HFO-1234yf ranged from 0.1 to 0.9. The calculated VLE properties were then compared with the experimental data by Ye et al.^58^

## Results and Discussion

### Pure Systems

To better understand the liquid structures, radial distribution functions *g*(*r*) (also called RDFs) of HFO-1234yf and HFO-1234ze­(E) were analyzed, as shown in Figure 2 and SI.

**Figure 2:**
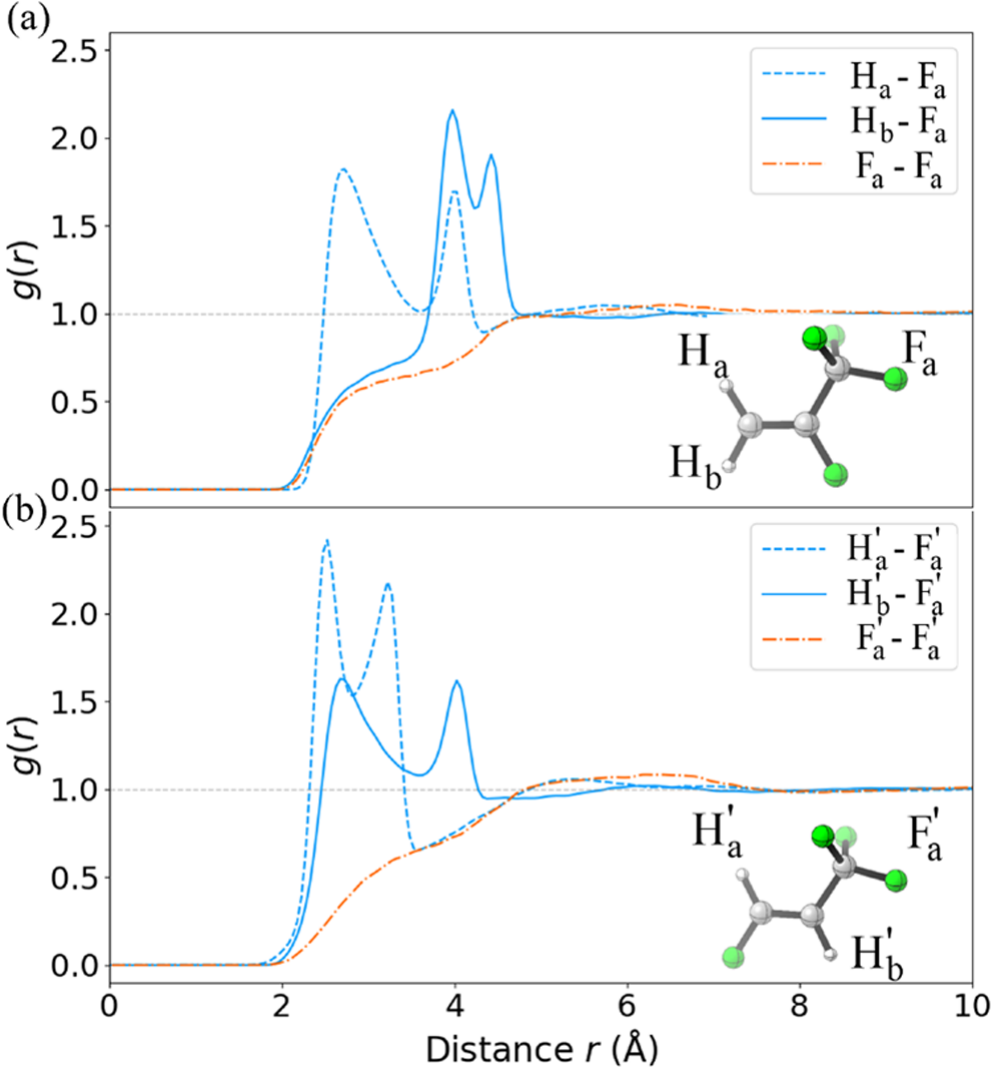
RDFs for selected H–F and F–F atom pairs in (a) HFO-1234yf and (b) HFO-1234ze­(E) in the liquid phase at 293.15 K and 10 bar.

The RDFs are consistent with those previously reported by Raabe and Maginn.^7,10^ In the intramolecular range, two distinct peaks are observed at 2–4 Å for HFO-1234yf and 3–5 Å for HFO-1234ze­(E), indicating the presence of hydrogen atoms in two distinct chemical environments in these compounds. In the intermolecular region, a shoulder peak from 5 to 6 Å indicates the presence of a weak hydrogen bond for HFO-1234yf and HFO-1234ze­(E). Additional RDFs for other pairs are provided in the SI.

The densities of the liquid and vapor phases were calculated as a function of the distance from the interface in the normal direction *z* and fitted to the tanh equation^59^

9 $$\rho \left(z\right)=\frac{1}{2}\left(\rho_{l}+\rho_{g}\right)-\frac{1}{2}\left(\rho_{l}-\rho_{g}\right)\tanh\left[\frac{2\left(z-z_{0}\right)}{d}\right]$$

where $\rho_{l}$ and ρ_g_ represent the densities of the liquid and gas phases, respectively. *z*_0_ = 0 denotes the position at which the density is half between $\rho_{l}$ and ρ_g_. *d* is related to the width of the liquid–vapor interface.

Snapshots of the HFO-1234yf simulations at three different temperatures, rendered using Visual Molecular Dynamics (VMD),^60^ are shown in Figure 3. As the temperature increases, more molecules enter the vapor phase, and the width of the liquid–vapor interface becomes wider, consistent with the density profiles in Figure 4.

**Figure 3:**
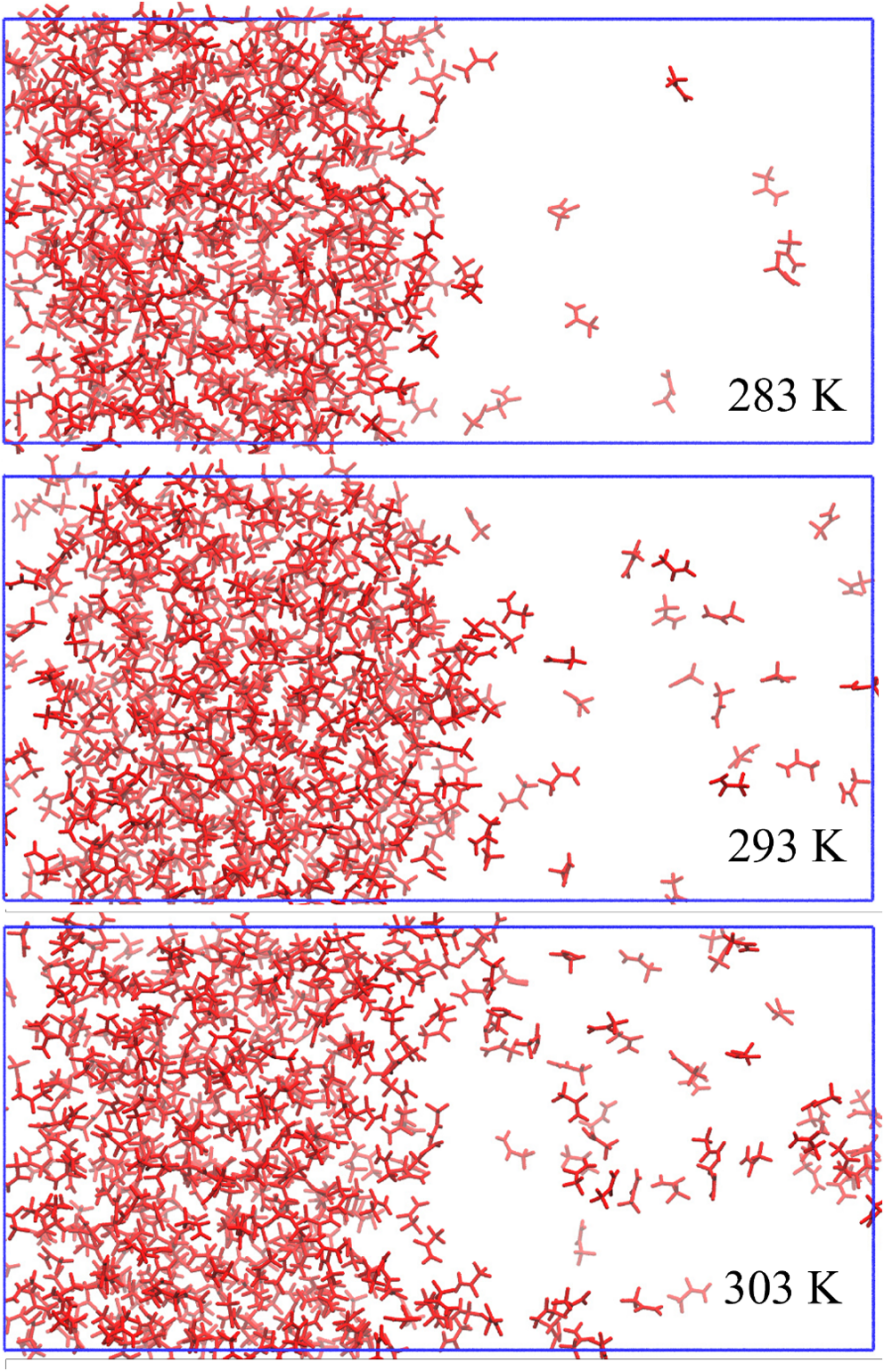
Representative snapshots of VLE simulations of HFO-1234yf at the indicated temperatures. HFO-1234yf molecules are shown in red.

**Figure 4:**
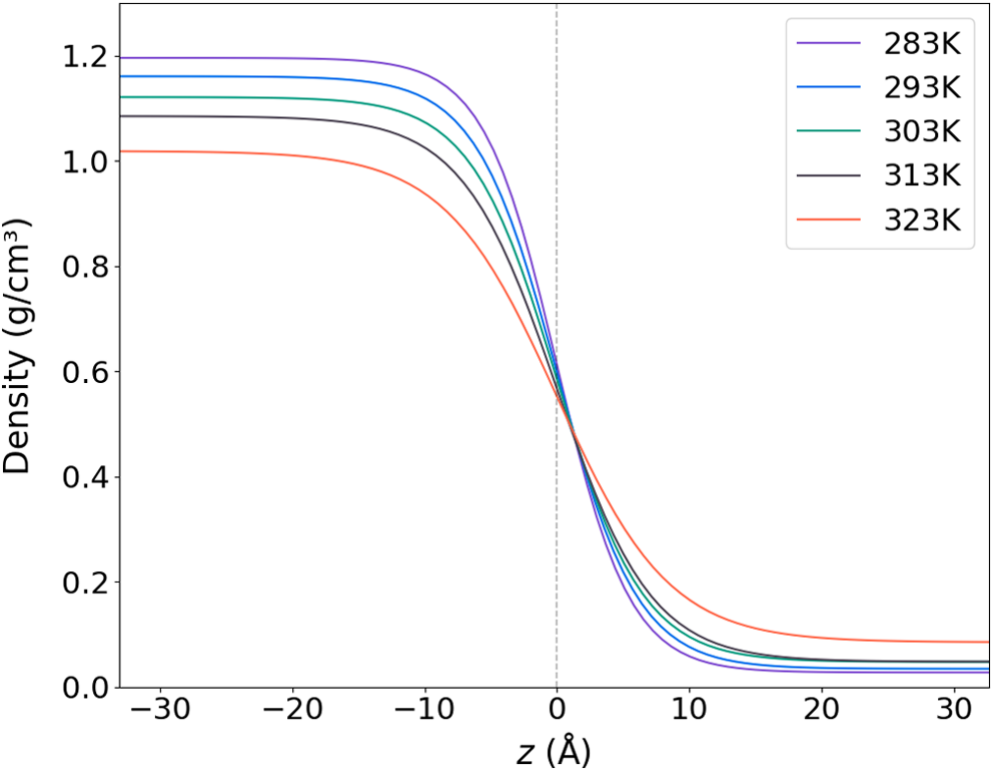
Fitted density profiles for HFO-1234yf at different temperatures along the *z*-axis with a bin width of 1.2 Å.

After having obtained the vapor densities for both components in the mixture at equilibrium, another constant NVT simulation was set up with those gas densities to calculate the total vapor pressure *P*_g_ to estimate the equilibrium saturation pressure for the original VLE simulation. The results are shown in Figure 5. Although the model slightly underestimates the liquid density, it successfully captures the overall decreasing trend as temperature increases. Additionally, the predicted saturated vapor densities and pressures closely match the experimental values, with minimal deviation, indicating that the model accurately represents the system’s behavior in the vapor phase.

**Figure 5:**
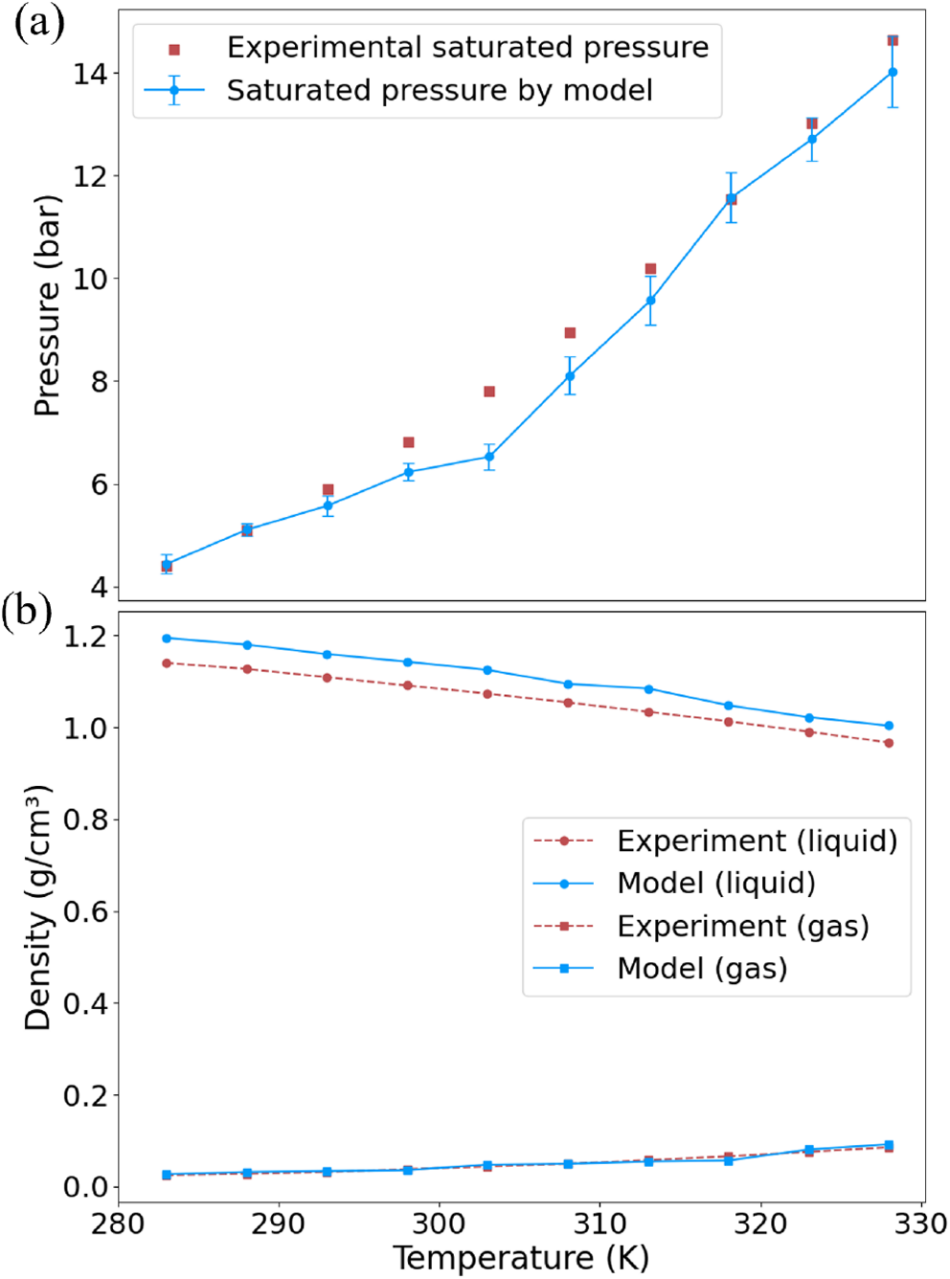
(a) Comparison of saturated vapor pressures at different temperatures and (b) liquid and vapor densities of HFO-1234yf.

The critical temperature *T*_c_ and the density ρ_c_ were extrapolated using the density scaling law^61^

10 $$\rho_{l}-\rho_{g}=A{\left(T_{c}-T\right)}^{\beta }$$

and the law of rectilinear diameters^62^

11 $$\frac{1}{2}\left(\rho_{l}+\rho_{g}\right)=\rho_{c}+B\left(T_{c}-T\right)$$

where *A* and *B* are fitting parameters and β is the critical exponent, typically taken as 0.325.^62^ The critical pressure *P*_c_ can be estimated using the Rackett equation^63,64^

12 $$\ln P_{c}=\ln\left(\frac{V_{l}}{V_{c}}\right)\cdot {\left(1-\frac{T}{T_{c}}\right)}^{-2/7}-\ln\left(\frac{V_{c}}{RT_{c}}\right)$$

where *R* is the ideal gas constant, $V_{l}$ represents the molar volume of the liquid at temperature *T*, *V*_c_ denotes the molar liquid volume at *P*_c_ and *T*_c_.

For the design of an AC system, the VLE properties of the refrigerant are crucial in determining the size of AC system and operating temperature range. Thermodynamic properties such as the enthalpy of vaporization Δ*H*_vap_ and isobaric heat capacity *C*_P_ directly influence the overall performance and efficiency of the system. In general, they are vital when considering candidate refrigerant mixtures for specific applications. The predicted Δ*H*_vap_ and *C*_P_ for HFO-1234yf, shown in Figure 6, demonstrate that our model can reasonably reproduce these properties.^40,41^

**Figure 6:**
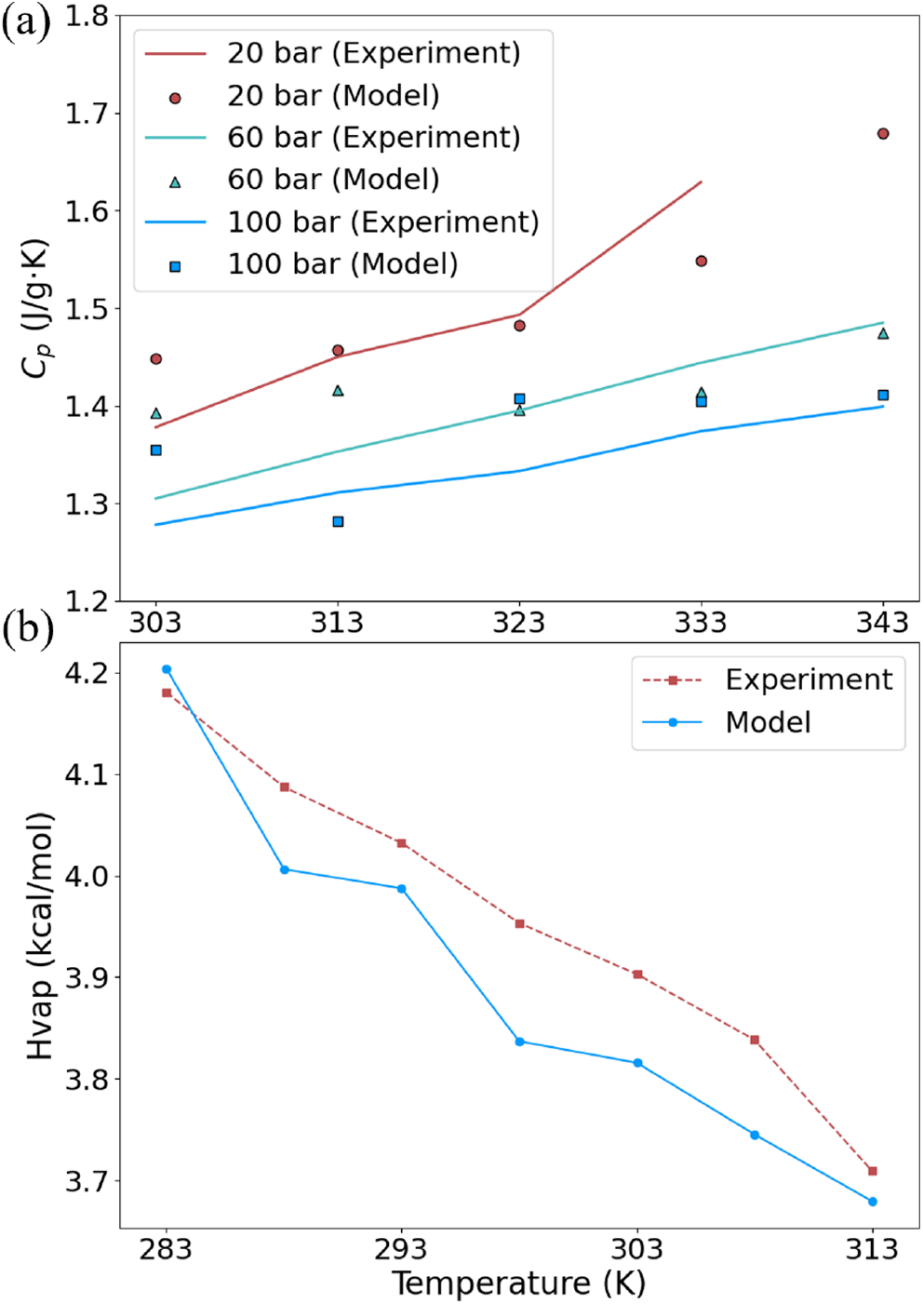
Comparison of (a) isobaric heat capacity as a function of temperature at three pressures. Mean absolute percentage errors (MAPEs) were 2.8% at 20 bar, 2.8% at 60 bar, and 3.4% at 100 bar and (b) enthalpy of vaporization at saturation pressure, with a MAPE of 1.7%. Note that experimental heat capacity data at 20 bar are not available above 343 K. See the SI for more details.

In addition, the results for HFO-1234yf/HFO-1234ze­(E) summarized in Table 1 demonstrate that our model exhibits good extrapolative capability and can reliably extrapolate thermophysical properties from room temperature up to the critical temperature.

**Table 1:** Critical Properties of HFO-1234yf and HFO-1234ze­(E)^65,66^

	HFO-1234yf		HFO-1234ze(E)	
critical point properties	experiment	model	experiment	model
temperature (K)	367.85	365.68	382.55	379.74
density (g/cm^3^)	0.475	0.492	0.489	0.492
pressure (bar)	33.81	33.43	36.36	34.91

For HFO-1234ze­(E), similar qualitative agreements as those for HFO-1234yf were observed. These HFO-1234ze­(E) results are shown in the SI (Figure S3).

### Binary Systems

As discussed in the introduction, predicting properties and understanding the physical behavior of multicomponent HFO systems is important in the study of refrigerants. To evaluate the accuracy of the force field for mixtures, VLE properties were calculated as functions of the temperature and mixture composition, as shown below. The numerical data are also available in Table S11 in the SI.

A critical aspect of designing efficient cooling systems is selecting the appropriate liquid- and vapor-phase compositions to ensure optimal performance. The chemical compositions of refrigerants govern their density and pressure–temperature relationship, which are the primary inputs for compressor design in the cooling cycle. Therefore, it is worthwhile to accurately determine the equilibrium composition of the liquid and vapor phases.

The density profile of each component in the binary system was fitted using eq 9 to determine their densities in the liquid and vapor phases. The resulting mole fractions were then calculated and compared with the values reported by Ye et al.^58^ as shown in Figure 7. Also, compared to the approximate experimental saturated liquid density (1.146 g/cm^3^) by Al Ghafri et al.^44^ for a 50:50 molar mixture at 293 K and 1 atm, the predicted value by our model at 1.173 g/cm^3^ has a relative error smaller than 3%. As illustrated by the VLE snapshot in Figure 8, HFO-1234yf exhibits a greater tendency to escape into the vapor phase.

**Figure 7:**
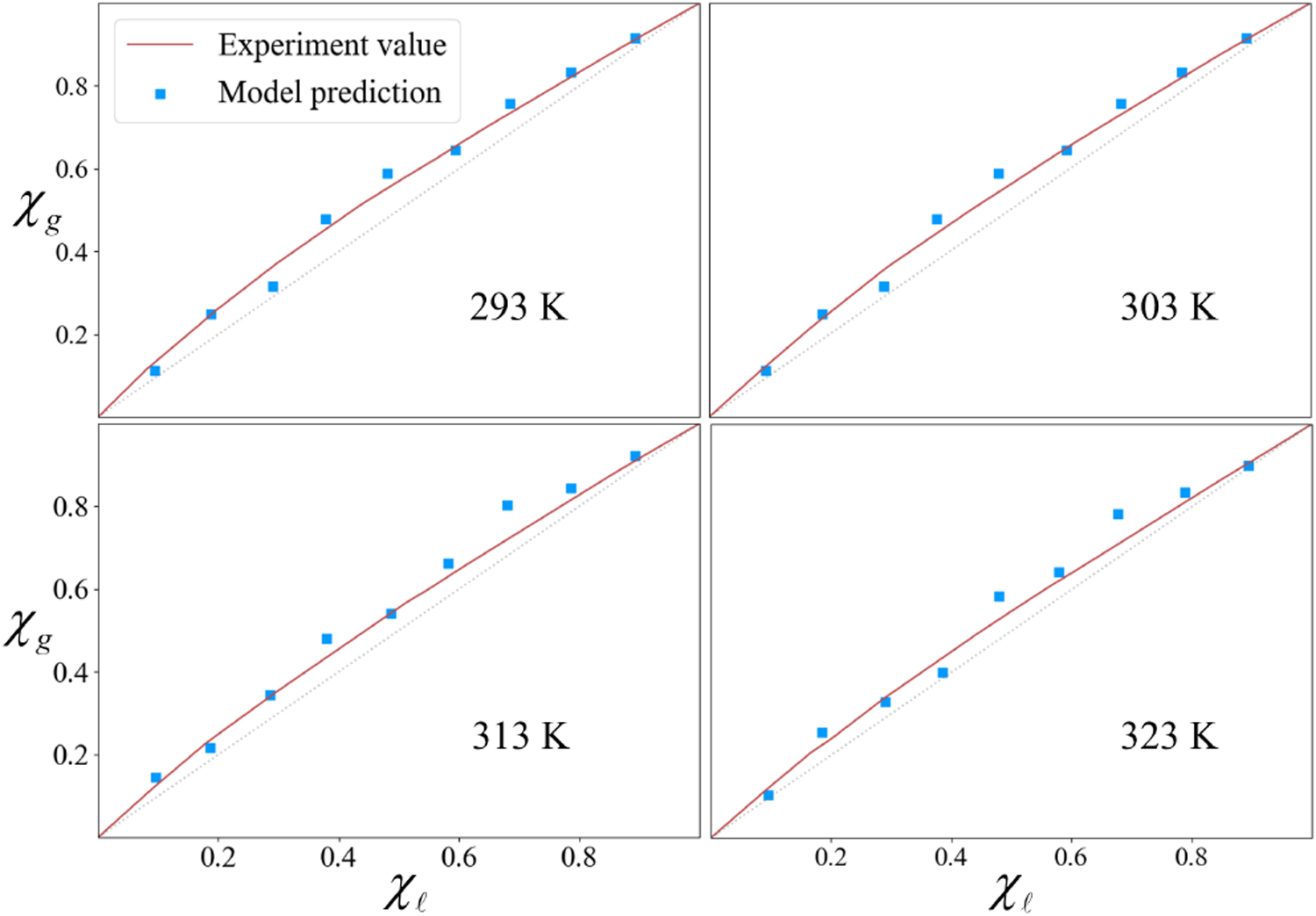
Equilibrium mole fractions of HFO-1234yf in the liquid phase $\chi_{l}$ and vapor phase χ_g_ at different temperatures.

**Figure 8:**
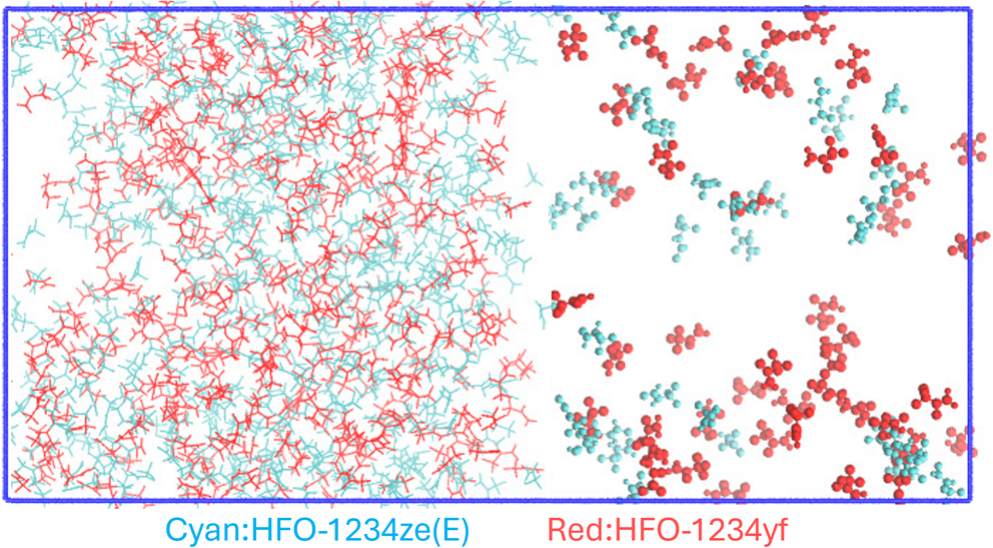
Snapshot of VLE simulations of the equimolar binary mixture at 343 K, HFO-1234yf and HFO-1234ze­(E) molecules are shown in red and cyan, respectively.

At a given temperature, refrigerants with higher saturation pressure often exhibit higher vapor density. This means a smaller volume of gas can carry more heat, enabling more compact compressors that are better suited to residential applications. Using the same method as in Figure 5, the vapor pressures were calculated and compared with the experimental values, as shown in Figure 9. The mean absolute error (MAE) is only about 0.43 bar.

**Figure 9:**
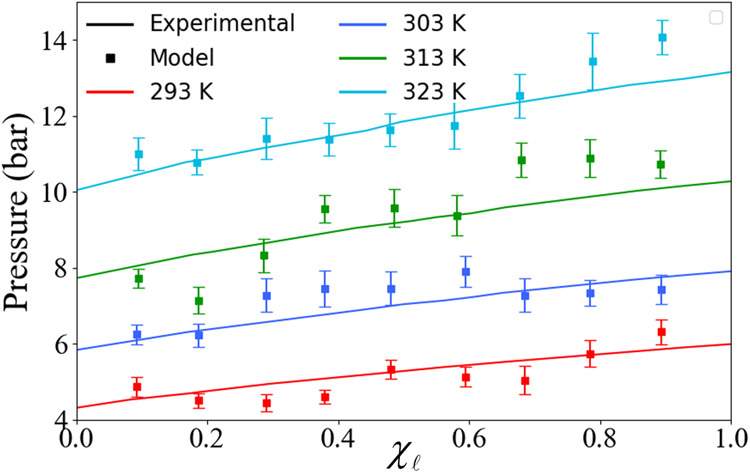
Comparison of saturation pressure between experimental measurements and model predictions as a function of liquid-phase HFO-1234yf mole fraction $\chi_{l}$ at different temperatures. As suggested in Figure 8, $\chi_{l}$ differs from the χ in the gas phase. Error bars were obtained from 20 independent simulations.

Surface tension plays a crucial role in droplet formation and can significantly influence the efficiency of heat exchangers. Therefore, we calculated the surface tensions^67^ σ predicted by our models by using the diagonal terms of the pressure tensor *P*_*xx*_, *P*_*yy*_, and *P*_*zz*_ obtained from the MD simulations.

13 $$\sigma =\frac{L_{z}}{2}\left(P_{\mathrm{zz}}-\frac{P_{\mathrm{xx}}+P_{\mathrm{yy}}}{2}\right)$$

where *L*_*z*_ is the simulation box length in the *z* dimension.

The surface tensions of both the pure HFOs and the equimolar mixture were calculated as a function of the temperature, as shown in Figure 10. Our models can reasonably reproduce the surface tensions with an MAE of 1.36 mN/m.

**Figure 10:**
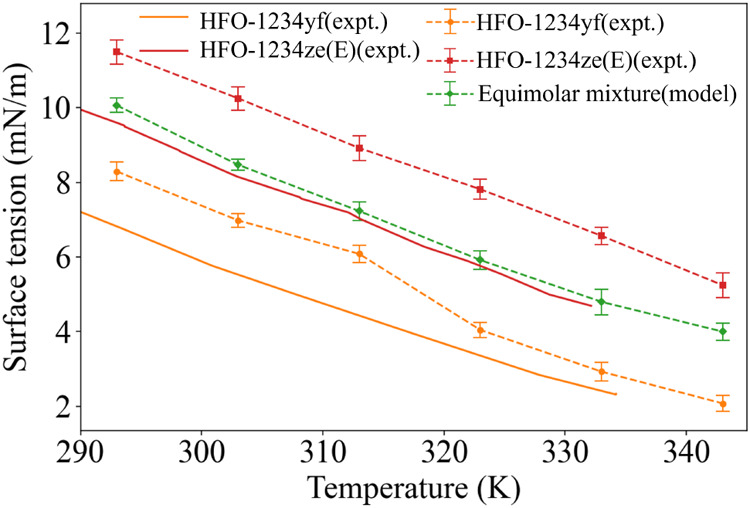
Comparison of the experimental and calculated surface tension as a function of temperature for the pure HFOs and the equimolar HFO-1234yf/HFO-1234ze­(E) mixture.^65,68^ Error bars were obtained from 20 independent simulations.

To analyze the key molecular interactions in the refrigerants, an energy decomposition analysis of the LJ interactions was performed for the equimolar binary mixture (Table S12). The average LJ energy between a pair of HFO-1234yf molecules (A–A) is approximately −34.6 J/mol, whereas that between a pair of HFO-1234ze­(E) molecules (B–B) is slightly more negative at −35.5 J/mol. Therefore, the LJ attraction is slightly stronger for B–B than for A–A interactions. This trend is further supported by a thermodynamic integration calculation at 343 K. For the alchemical exchange process in which one HFO-1234yf molecule dissolves into the solution while another HFO-1234ze­(E) molecule evaporates, the calculated free energy change is +2.58 kJ/mol (see SI for more details). The positive value indicates a greater preference for HFO-1234yf to stay in the gas phase, leading to its enrichment in the vapor phase. This observation aligns with its lower normal boiling point.^38,40^ of HFO-1234yf (−29.5 °C) than that of HFO-1234ze­(E) (−19.0 °C) and also consistent with positive deviation of HFO-1234yf from Raoult’s law.

## Conclusions

In this work, we developed a data-efficient strategy for reparameterizing classical polarizable force fields for low-GWP hydrofluoroolefin refrigerants, focusing on HFO-1234yf and HFO-1234ze­(E). By combining high-level DFT potential-energy data with a limited set of condensed-phase experimental properties, including liquid density, isobaric heat capacity, and enthalpy of vaporization, the resulting models achieve reliable accuracy for both pure fluids and their binary mixtures.

For the pure fluids, the optimized force fields reproduce liquid and vapor densities, saturation pressures, isobaric heat capacities, and enthalpies of vaporization over broad temperature ranges, with most deviations from experiment within 5%. The models also predict critical properties in good agreement with experiment, demonstrating their ability to extrapolate toward near-critical conditions. For binary mixtures, the optimized cross-interaction parameters capture vapor–liquid equilibrium behavior across HFO-1234yf mole fractions from 0.1 to 0.9 and temperatures from 293 to 343 K. The predicted saturation pressures show close agreement with experiment, with a mean absolute error of 0.43 bar. The models also reproduce surface tensions of the pure fluids and equimolar mixture with a mean absolute error of 1.36 mN/m, highlighting their capability to describe explicit liquid–vapor interfaces. Consistent with these macroscopic trends, energy decomposition and thermodynamic integration analyses show that HFO-1234yf preferentially partitions into the vapor phase, in agreement with its lower normal boiling point and positive deviation from Raoult’s law.

Overall, this framework provides an accurate and transferable molecular simulation route for predicting thermophysical properties, interpreting nonideal mixing, and screening next-generation low-GWP refrigerant blends.

## Supplementary Material



## Data Availability

Initial molecular configurations were generated using PACKMOL 21.1.1 (https://m3g.github.io/packmol/). Cluster searches were performed with the assistance of Molclus 1.12 (http://www.keinsci.com/research/molclus.html) and xtb 6.7.1 (https://xtb-docs.readthedocs.io/en/latest/) prior to quantum mechanical calculations. Geometry optimizations were carried out using Gaussian 16, Revision C.02 (https://gaussian.com/), and single-point energy calculations were performed using ORCA 6.0.0 (https://www.faccts.de/orca/). Molecular dynamics simulations were conducted using OpenMM 8.1.0 (https://openmm.org/). Molecular structures were rendered with Visual Molecular Dynamics (VMD 1.9.4) (https://www.ks.uiuc.edu/Research/vmd/). Figures and plots were prepared using the Python library Matplotlib 3.10.9 (https://matplotlib.org/). All data required to reproduce this study, including topologies, input files, coordinates, and analysis scripts, have been deposited in Zenodo (10.5281/zenodo.20115825).

## Notes

Force field parameters, RDF figures, thermodynamic and VLE properties for the pure and mixture systems, and free energy calculation details (PDF)
