# Supplementary material for: Developing Efficient and Accurate Polarizable Molecular Simulation Models for Studying Refrigerants and Their Binary Mixtures
Source: J Phys Chem B. 2026 Jul 22;130(31):8052–60. doi: 10.1021/acs.jpcb.6c03275 (PMC13455069; doi:10.1021/acs.jpcb.6c03275)
Supplement: Supplementary file 1 [file jp6c03275_si_001.pdf]

Developing Efficient and Accurate Polarizable Molecular Simulation Models for Studying Refrigerants and Their Binary Mixtures

Haihui WANG, Ying-Lung Steve Tse*

Department of Chemistry, The Chinese University of Hong Kong, Shatin, NT, Hong Kong

Email: stevetse@cuhk.edu.hk

Force Field Parameters of the Optimized Models (Figure S1, Tables S1-S5)

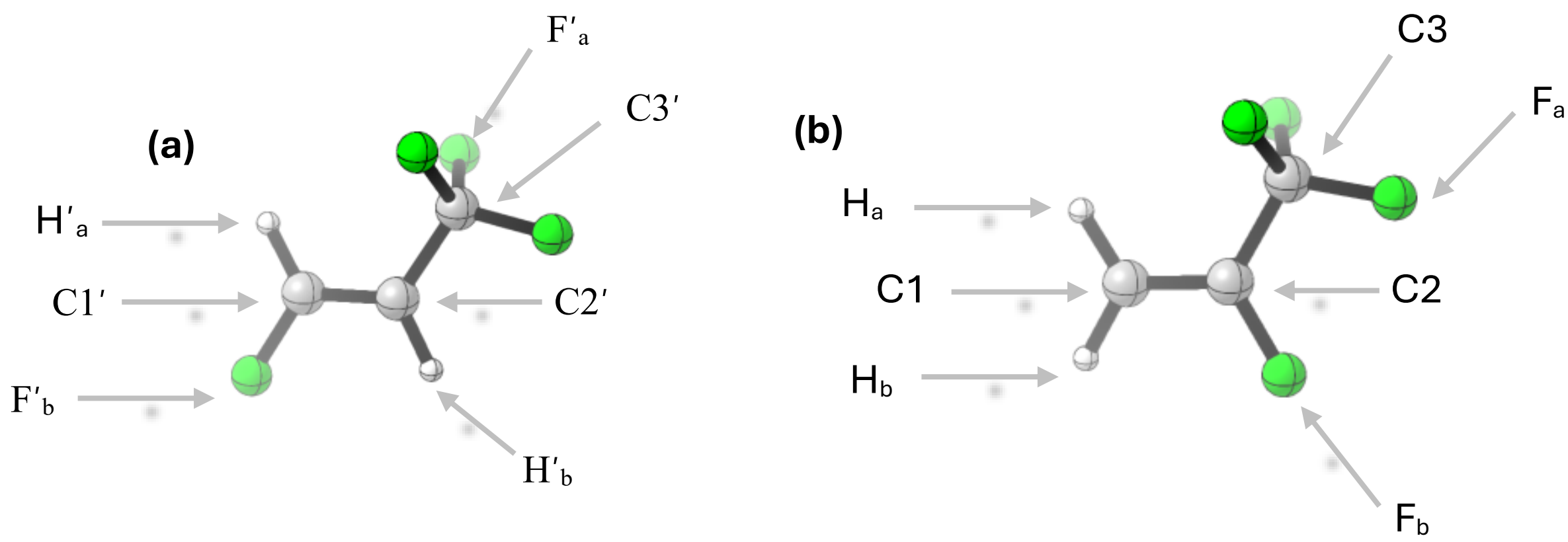

Figure S1. Molecular structures and atom types of (a) HFO-1234ze(E) and (b) HFO-1234yf.

Table S1. Atomic masses *M*, partial charges *Q*, polarizability volumes α' and scaling factors THOLE of different atom types of the optimized models. The polarizability α is given by $\alpha = 4\pi\varepsilon_0\alpha'$ and the Drude charges q_D are given by $q_D = \sqrt{k_D\alpha}$.

Atom type	*M*	*Q*	α' / Å^3	THOLE
**HFO-1234ze(E)**				
C2′	12.010	-0.34680	-1.36248	1.220
C1′	12.010	0.17424	1.45140	1.312
F'_b	19.000	-0.16910	0.16579	1.314
C3′	12.010	0.71324	0.79432	1.292
F'_a	19.000	-0.18197	0.51582	1.320
H'_b	1.008	0.12210	/	/
H'_a	1.008	0.05223	/	/
**HFO-1234yf**				
C2	12.010	-0.06237	-1.11728	1.356
C1	12.010	-0.10813	-0.87125	1.360

F_b	19.000	-0.07313	-0.25409	1.342
C3	12.010	0.57333	-1.13431	1.291
F_a	19.000	-0.16730	-0.58108	1.308
H_a	1.008	0.07723	/	/
H_b	1.008	0.09497	/	/
**All Drude particles**				
DRUD	0.4000	/	/	/

Table S2. Bond parameters of the optimized models, where X is a wildcard.

Atom type 1	Atom type 2	k_b / kcal·mol^{-1}·Å^{-2}	r_0 / Å
**HFO-1234ze(E)**			
C1′	C2′	558.919	1.332050
C2′	C3′	323.296	1.520723
C2′	H'_b	298.778	1.085750
C1′	F'_b	331.698	1.334102
C1′	H'_a	299.316	1.075302
C3′	F'_a	302.553	1.346635
**HFO-1234yf**			
C1	C2	574.918	1.338436
C2	C3	324.934	1.496583
C2	F_b	374.303	1.343433
C1	H_b	345.493	1.093871
C1	H_a	343.650	1.094545
C3	F_a	353.425	1.345559
**All Drude oscillators**			
X	DRUD	k_D = 1000.000	0.000000

Table S3. Angle parameters of the optimized models.

Atom type 1	Atom type 2	Atom type 3	k_a / kcal·mol^{-1}·rad^{-2}	θ_0 / deg
**HFO-1234ze(E)**				
C2′	C1′	F′$_b$	65.429	121.16542
C2′	C1′	H′$_a$	42.417	122.94113
C2′	C3′	F′$_a$	74.493	110.52849
C1′	C2′	C3′	60.196	123.52413
C1′	C2′	H′$_b$	42.234	119.54503
F′$_b$	C1′	H′$_a$	57.603	112.33108
C3′	C2′	H′$_b$	52.789	115.60354
F′$_a$	C3′	F′$_a$	67.047	107.99538
**HFO-1234yf**				
C2	C1	H$_b$	49.355	119.77016
C2	C1	H$_a$	50.015	120.35586
C2	C3	F$_a$	66.065	111.74324
C1	C2	C3	64.026	122.62506
C1	C2	F$_b$	67.439	123.33699
H$_b$	C1	H$_a$	38.069	117.60741
C3	C2	F$_b$	65.683	112.21639
F$_a$	C3	F$_a$	70.548	106.56075

Table S4. Proper and improper torsion parameters of the optimized models.

**Proper torsion**						
Atom type 1	Atom type 2	Atom type 3	Atom type 4	k_d / kcal·mol^{-1}	n	γ / deg
**HFO-1234ze(E)**						
C3′	C2′	C1′	F′$_b$	6.65	2	180
H′$_b$	C2′	C1′	H′$_a$	6.65	2	180
**HFO-1234yf**						
C3	C2	C1	H$_b$	6.65	2	180
F$_b$	C2	C1	H$_a$	6.65	2	180
**Improper torsion**						
**HFO-1234ze(E)**						
C1′	C3′	C2′	H′$_b$	1.1	2	180
C1′	F′$_b$	C2′	H′$_a$	1.1	2	180
**HFO-1234yf**						
C1	C3	C2	F$_b$	1.1	2	180
C1	H$_b$	C2	H$_a$	1.1	2	180

Table S5. Lennard-Jones parameters of the optimized models, where X is a wildcard.

Atom type 1	Atom type 2	ε / kcal·mol^{-1}	σ / Å
	**HFO-1234ze(E) ··· HFO-1234ze(E)**		
C1′	C1′	-0.079717	3.013009
C1′	C2′	-0.084553	4.211763
C1′	F′$_b$	-0.070826	3.735041
C1′	F′$_a$	-0.072545	3.687260
C1′	C3′	-0.118602	3.927054
C1′	H′$_b$	-0.045525	3.510933
C1′	H′$_a$	-0.034184	4.023992
C2′	C2′	-0.061370	3.594636
C2′	F′$_b$	-0.088027	3.587985
C2′	F′$_a$	-0.090482	3.437814
C2′	C3′	-0.086920	3.684448
C2′	H′$_b$	-0.046944	3.155154
C2′	H′$_a$	-0.031398	3.335754
F′$_b$	F′$_b$	-0.063114	3.252433
F′$_b$	F′$_a$	-0.050390	2.647029
F′$_b$	C3′	-0.077562	2.217230
F′$_b$	H′$_b$	-0.022067	3.139923
F′$_b$	H′$_a$	-0.042160	2.390512
F′$_a$	F′$_a$	-0.069065	1.748236
F′$_a$	C3′	-0.108297	3.954729
F′$_a$	H′$_b$	-0.035555	2.111559
F′$_a$	H′$_a$	-0.027860	2.772921
C3′	C3′	-0.103483	3.173698
C3′	H′$_b$	-0.036479	4.170350
C3′	H′$_a$	-0.030804	2.911602
H′$_b$	H′$_b$	-0.015124	2.853395
H′$_b$	H′$_a$	-0.010769	3.384127
H′$_a$	H′$_a$	-0.016410	2.014144
	**HFO-1234yf ··· HFO-1234yf**		
C1	C1	-0.097691	4.041886
C1	C2	-0.082366	3.853896
C1	F$_b$	-0.086890	3.641836
C1	F$_a$	-0.065253	3.505922
C1	C3	-0.098573	4.354685
C1	H$_b$	-0.041970	3.382818
C1	H$_a$	-0.031836	3.641352
C2	C2	-0.103190	3.703537
C2	F$_b$	-0.071258	3.326676
C2	F$_a$	-0.063543	3.636844
C2	C3	-0.089078	3.142346
C2	H$_b$	-0.042201	3.724105

C2	H_a	-0.041544	2.799740
F_b	F_b	-0.072584	2.401759
F_b	F_a	-0.063092	3.066141
F_b	C3	-0.100768	2.969066
F_b	H_b	-0.040779	2.670001
F_b	H_a	-0.029750	2.095335
F_a	F_a	-0.082229	2.742316
F_a	C3	-0.061626	3.818025
F_a	H_b	-0.037150	3.257277
F_a	H_a	-0.039511	2.849989
C3	C3	-0.114605	3.344254
C3	H_b	-0.046963	3.296493
C3	H_a	-0.048313	3.813746
H_b	H_b	-0.013919	2.824720
H_b	H_a	-0.016881	2.433217
H_a	H_a	-0.017757	2.965012
**HFO-1234ze(E) ··· HFO-1234yf**			
C1′	C1	-0.106187	4.494011
C1′	C2	-0.110923	3.983720
C1′	F_b	-0.067022	2.564476
C1′	F_a	-0.085900	3.838788
C1′	C3	-0.074404	4.166394
C1′	H_b	-0.044094	2.961222
C1′	H_a	-0.038052	3.072457
C2′	C1	-0.065377	3.102177
C2′	C2	-0.102648	4.539251
C2′	F_b	-0.078269	2.659579
C2′	F_a	-0.084157	3.665036
C2′	C3	-0.104032	2.702395
C2′	H_b	-0.034699	3.943481
C2′	H_a	-0.038028	2.328106
F'_b	C1	-0.050702	2.983661
F'_b	C2	-0.063212	2.597414
F'_b	F_b	-0.055961	2.484861
F'_b	F_a	-0.052030	2.530743
F'_b	C3	-0.072820	3.729830
F'_b	H_b	-0.035230	3.645697
F'_b	H_a	-0.021405	2.984062
F'_a	C1	-0.051762	2.712352
F'_a	C2	-0.069644	2.905033
F'_a	F_b	-0.060273	3.085775
F'_a	F_a	-0.050692	2.988610
F'_a	C3	-0.092938	3.824582
F'_a	H_b	-0.022228	2.890283
F'_a	H_a	-0.033568	3.303202

C3′	C1	-0.090273	4.338593
C3′	C2	-0.072520	4.753655
C3′	F_b	-0.074473	2.906125
C3′	F_a	-0.088087	3.243896
C3′	C3	-0.112038	4.741175
C3′	H_b	-0.052350	4.157303
C3′	H_a	-0.030480	3.574258
H'_b	C1	-0.028372	3.900985
H'_b	C2	-0.033784	4.193161
H'_b	F_b	-0.038509	2.309630
H'_b	F_a	-0.021209	2.639503
H'_b	C3	-0.050815	4.336708
H'_b	H_b	-0.018941	2.734264
H'_b	H_a	-0.019418	3.023321
H'_a	C1	-0.037778	2.894756
H'_a	C2	-0.032739	3.086855
H'_a	F_b	-0.025962	2.262319
H'_a	F_a	-0.028973	3.057527
H'_a	C3	-0.029062	4.057935
H'_a	H_b	-0.012403	2.525470
H'_a	H_a	-0.011686	3.538428
X	DRUD	0.000000	0.000000

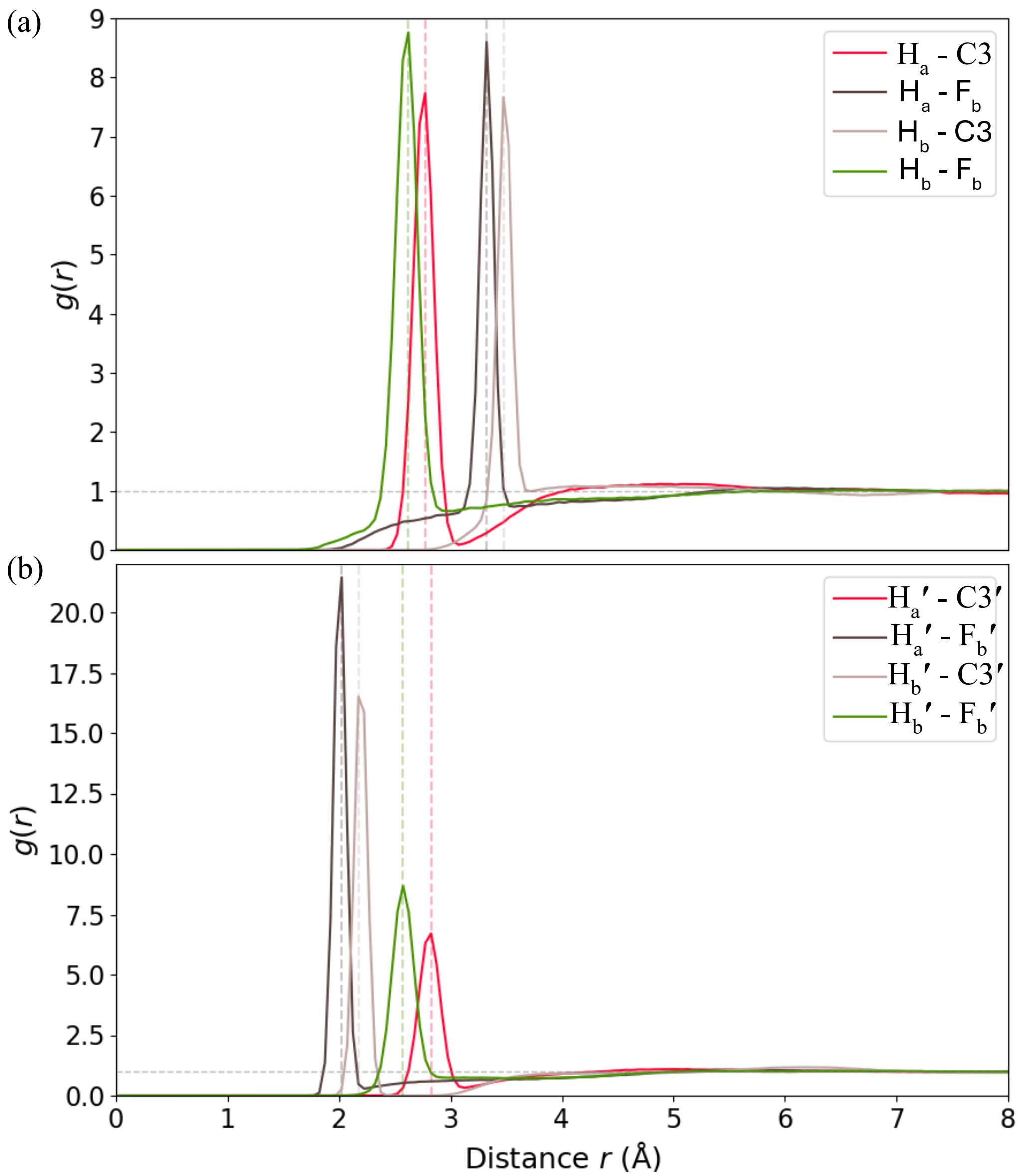

Figure S2. Radial distribution functions (RDFs, $g(r)$) for selected atomic pairs of (a) HFO-1234yf and (b) HFO-1234ze(E) in the liquid phase at 293.15 K and 10 bar. Vertical dotted lines indicate the peak positions.

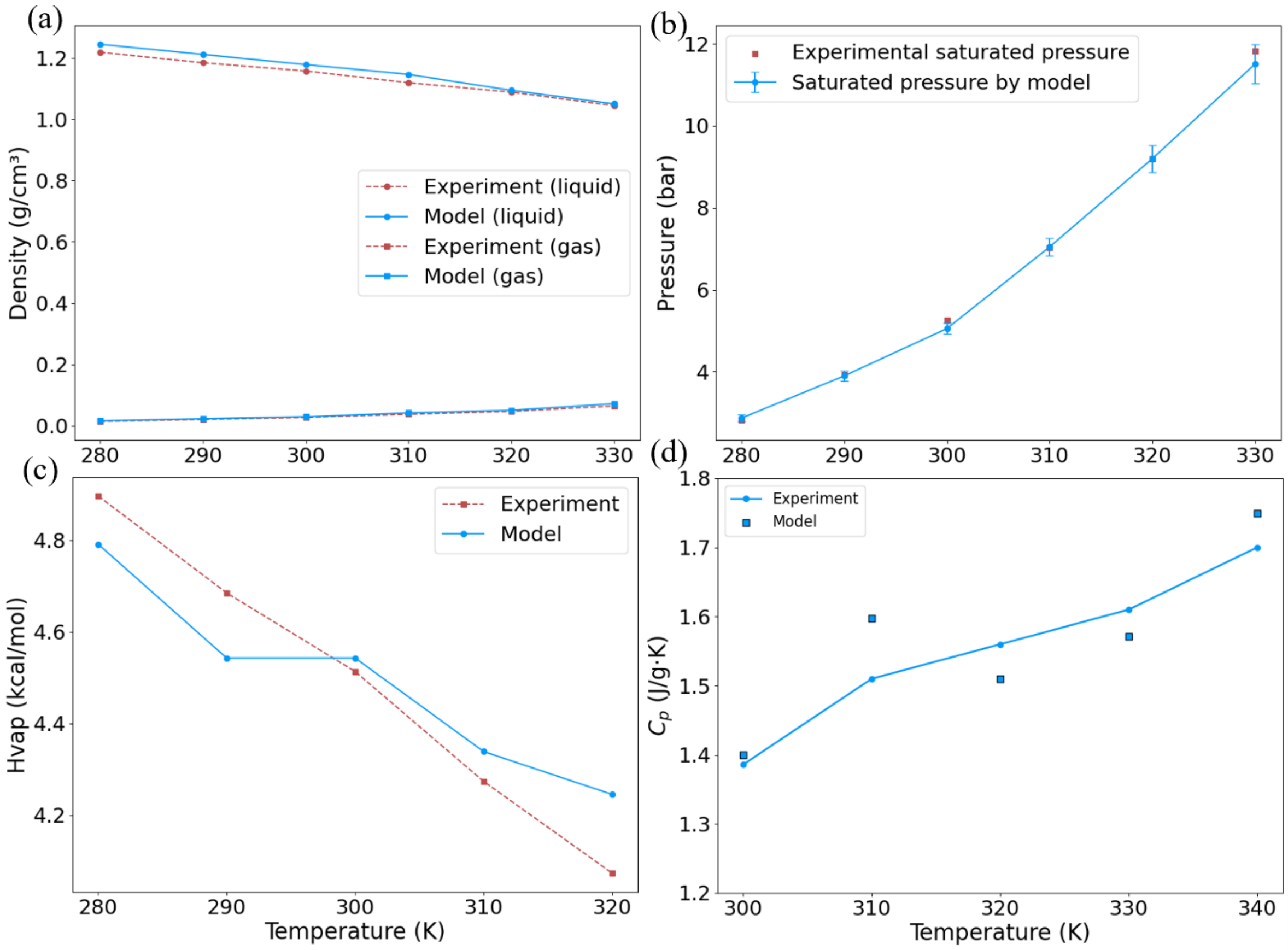

Figure S3. Comparison of (a) saturated liquid and vapor densities, (b) saturated vapor pressure, (c) heat of evaporation, and (d) isobaric heat capacity under saturation conditions for HFO-1234ze(E).

Table S6. VLE properties of HFO-1234yf predicted by the optimized model. The standard errors were estimated from 20 independent simulation runs.

**Temperature** / K	**liquid density** / g $\cdot$cm^{-3}	**Vapor density** / g $\cdot$cm^{-3}	**Saturated pressure** / bar	**Std. error of pressure** / bar
283	1.194	0.027	4.438	0.185
288	1.180	0.032	5.105	0.116
293	1.159	0.034	5.573	0.198
298	1.143	0.036	6.227	0.169
303	1.125	0.048	6.525	0.248
308	1.095	0.05	8.108	0.366
313	1.084	0.055	9.570	0.476
318	1.048	0.077	11.570	0.485
323	1.022	0.081	12.714	0.424
328	1.003	0.092	14.026	0.691

Table S7. Comparison of experimental and model-predicted heat of evaporation for HFO-1234yf ($kcal \cdot mol^{-1}$).

**Temperature / K**	Experiment	Model
283	4.180	4.203
288	4.087	4.006
293	4.032	3.987
298	3.953	3.837
303	3.903	3.816
308	3.838	3.745
313	3.709	3.680

Table S8. Comparison of experimental and model-predicted isobaric heat capacities of HFO-1234yf at different temperatures and three different pressures ($J \cdot g^{-1} \cdot K^{-1}$).

**Temperature** / K	**Pressure** = 20 bar		**Pressure** = 60 bar		**Pressure** = 100 bar	
	Experiment	Model	Experiment	Model	Experiment	Model
283	/	1.317	/	1.296	/	1.344
293	/	1.409	/	1.378	/	1.407
303	1.378	1.448	1.305	1.393	1.278	1.355
313	1.450	1.457	1.401	1.416	1.353	1.282
323	1.493	1.482	1.445	1.396	1.333	1.407
333	1.629	1.549	1.538	1.414	1.374	1.405
343	/	1.679	1.485	1.475	1.399	1.411
353	/	1.135	1.601	1.619	1.455	1.413

Table S9. VLE properties of HFO-1234ze(E) predicted by the optimized model. Standard errors were estimated from 20 independent simulation runs.

**Temperature** / K	**liquid density** / $g \cdot cm^{-3}$	**Vapor density** / $g \cdot cm^{-3}$	**Saturated Pressure** / bar	**Std. error of pressure** / bar
280	1.244	0.017	2.870	0.079
290	1.211	0.023	3.900	0.127
300	1.178	0.030	5.054	0.135
310	1.146	0.043	7.046	0.208
320	1.094	0.051	9.200	0.327
330	1.050	0.072	11.506	0.479

Table S10. Comparison of experimental and model-predicted heat of evaporation and isobaric heat capacities for HFO-1234ze(E) under saturation conditions.

**Heat of evaporation** / kcal·mol^{-1}			**Heat capacity** / J·g^{-1}·K^{-1}		
**Temperature** / K	Experiment	Model	**Temperature** / K	Experiment	Model
280	4.897	4.792	300	1.386	1.399
290	4.685	4.543	310	1.510	1.598
300	4.514	4.543	320	1.560	1.509
310	4.273	4.339	330	1.610	1.571
320	4.073	4.245	340	1.700	1.749

Table S11. VLE properties of the binary mixture of HFO-1234yf and HFO-1234ze(E) predicted by the optimized model. x and y represent the mole fractions of HFO-1234yf in the liquid and vapor phases, respectively. The pressure values and their standard errors were estimated from 20 independent simulation runs.

**Temperature** / K	**Liquid density** / g ·cm^{-3}	**Vapor density** / g ·cm^{-3}	x	y	**Saturated pressure** / bar	**Std. error of pressure** / bar
293	1.214	0.026	0.088	0.181	4.868	0.253
293	1.197	0.024	0.191	0.241	4.505	0.199
293	1.190	0.023	0.287	0.354	4.439	0.229
293	1.178	0.025	0.387	0.500	4.601	0.189
293	1.174	0.028	0.489	0.533	5.322	0.247
293	1.166	0.028	0.582	0.691	5.127	0.262
293	1.163	0.027	0.688	0.726	5.042	0.376
293	1.160	0.031	0.790	0.842	5.744	0.352
293	1.161	0.034	0.891	0.959	6.311	0.338
303	1.183	0.032	0.093	0.113	6.247	0.262
303	1.175	0.032	0.187	0.249	6.227	0.307
303	1.156	0.038	0.290	0.315	7.281	0.433
303	1.143	0.038	0.379	0.477	7.455	0.469
303	1.141	0.038	0.481	0.589	7.460	0.445
303	1.137	0.041	0.594	0.645	7.911	0.404
303	1.130	0.038	0.685	0.758	7.279	0.447
303	1.128	0.038	0.785	0.834	7.336	0.340
303	1.127	0.045	0.893	0.916	7.431	0.386
313	1.147	0.039	0.095	0.145	7.718	0.247
313	1.135	0.035	0.187	0.217	7.146	0.341
313	1.124	0.041	0.286	0.344	8.322	0.433
313	1.111	0.047	0.380	0.481	9.552	0.368
313	1.106	0.048	0.486	0.541	9.569	0.496

313	1.096	0.047	0.582	0.663	9.373	0.531
313	1.095	0.054	0.680	0.804	10.849	0.449
313	1.091	0.054	0.785	0.845	10.894	0.494
313	1.090	0.053	0.892	0.921	10.735	0.357
323	1.108	0.051	0.095	0.104	11.010	0.429
323	1.100	0.052	0.184	0.255	10.784	0.336
323	1.085	0.054	0.290	0.328	11.401	0.542
323	1.077	0.054	0.386	0.401	11.384	0.440
323	1.066	0.056	0.479	0.585	11.634	0.424
323	1.059	0.057	0.578	0.643	11.756	0.616
323	1.049	0.061	0.677	0.783	12.526	0.569
323	1.048	0.067	0.788	0.836	13.449	0.743
323	1.049	0.068	0.895	0.901	14.070	0.447
333	1.072	0.069	0.1	0.11	14.7	0.822
333	1.061	0.072	0.192	0.207	15.666	0.798
333	1.047	0.061	0.289	0.321	14.456	0.719
333	1.036	0.08	0.384	0.428	16.85	1.003
333	1.018	0.077	0.471	0.566	16.458	0.54
333	1.018	0.078	0.584	0.639	17.025	0.805
333	1.003	0.08	0.683	0.75	17.044	0.823
333	1.005	0.096	0.785	0.838	20.461	0.966
333	0.995	0.103	0.89	0.924	22.358	0.923
343	1.041	0.073	0.096	0.120	15.889	0.692
343	1.008	0.092	0.187	0.241	16.935	0.921
343	1.005	0.088	0.285	0.328	17.201	0.888
343	0.984	0.073	0.390	0.424	18.921	0.702
343	0.972	0.090	0.476	0.591	19.551	0.928
343	0.968	0.117	0.581	0.632	25.69	0.966
343	0.946	0.103	0.693	0.729	22.732	1.132
343	0.949	0.113	0.785	0.827	25.019	0.786
343	0.941	0.127	0.894	0.911	27.639	1.445

Table S12. Pairwise decomposition of the intermolecular Lennard-Jones interaction energy of a 1000-molecule equimolar binary mixture.

Pair	Interaction energy (kJ/mol)	Pair	Interaction energy (kJ/mol)	Pair	Interaction energy (kJ/mol)
**HFO-1234yf···HFO-1234yf**					
C1-C1	-165.51	C2-C3	-76.95	F_a-H_b	-243.65
C1-C2	-232.54	C2-H_b	-103.44	F_a-H_a	-228.55
C1-F_b	-195.79	C2-H_a	-29.11	C3-C3	-69.77
C1-F_a	-388.25	F_b-F_b	-17.91	C3-H_b	-74.04
C1-C3	-432.25	F_b-F_a	-200.97	C3-H_a	-132.79
C1-H_b	-76.87	F_b-C3	-88.63	H_b-H_b	-6.48
C1-H_a	-64.11	F_b-H_b	-33.37	H_b-H_a	-7.70
C2-C2	-109.46	F_b-H_a	-9.32	H_a-H_a	-7.94
C2-F_b	-108.98	F_a-F_a	-274.74	/	/
C2-F_a	-395.94	F_a-C3	-438.31	/	/
Total			-4213.37		
Interaction energy per pair (J/mol)			-34.56		
**HFO-1234yf···HFO-1234ze(E)**					
$C1'$-C1	-460.88	F'_b-F_a	-75.64	$C3'$-H_a	-50.11
$C1'$-C2	-310.18	F'_b-C3	-152.97	H'_b-C1	-67.68
$C1'$-F_b	-43.28	F'_b-H_b	-75.23	H'_b-C2	-103.95
$C1'$-F_a	-669.41	F'_b-H_a	-4.04	H'_b-F_b	-12.88
$C1'$-C3	-275.83	F'_a-C1	-73.88	H'_b-F_a	-36.40
$C1'$-H_b	-33.05	F'_a-C2	-146.27	H'_b-C3	-172.17
$C1'$-H_a	-38.76	F'_a-F_b	-219.76	H'_b-H_b	-10.34
$C2'$-C1	-44.17	F'_a-F_a	-509.67	H'_b-H_a	-15.44
$C2'$-C2	-477.91	F'_a-C3	-753.64	H'_a-C1	-26.67
$C2'$-F_b	-44.70	F'_a-H_b	-47.18	H'_a-C2	-36.15
$C2'$-F_a	-539.44	F'_a-H_a	-117.25	H'_a-F_b	-4.48
$C2'$-C3	-33.39	$C3'$-C1	-346.64	H'_a-F_a	-94.73
$C2'$-H_b	-87.20	$C3'$-C2	-373.19	H'_a-C3	-89.79
$C2'$-H_a	-6.71	$C3'$-F_b	-63.83	H'_a-H_b	-4.98
F'_b-C1	-56.98	$C3'$-F_a	-374.86	H'_a-H_a	-20.40
F'_b-C2	-30.18	$C3'$-C3	-621.59	/	/
F'_b-F_b	-26.53	$C3'$-H_b	-162.76	/	/
Total			-8043.17		
Interaction energy per pair (J/mol)			-32.17		
**HFO-1234ze(E)···HFO-1234ze(E)**					

C1′-C1′	-32.62	C2′-C3′	-199.52	F'_{a}-H'_{b}	-28.80
C1′-C2′	-337.99	C2′-H'_{b}	-60.75	F'_{a}-H'_{a}	-174.68
C1′-F'_{b}	-159.61	C2′-H'_{a}	-47.40	C3′-C3′	-45.36
C1′-F'_{a}	-524.72	F'_{b}-F'_{b}	-45.77	C3′-H'_{b}	-101.77
C1′-C3′	-403.71	F'_{b}-F'_{a}	-159.79	C3′-H'_{a}	-26.09
C1′-H'_{b}	-94.35	F'_{b}-C3′	-34.51	H'_{b}-H'_{b}	-6.89
C1′-H'_{a}	-84.28	F'_{b}-H'_{b}	-14.21	H'_{b}-H'_{a}	-17.49
C2′-C2′	-60.75	F'_{b}-H'_{a}	-25.45	H'_{a}-H'_{a}	-0.92
C2′-F'_{b}	-213.41	F'_{a}-F'_{a}	-17.80	/	/
C2′-F'_{a}	-511.68	F'_{a}-C3′	-1003.48	/	/
Total			-4433.83		
Interaction energy per pair (J/mol)			-35.54		

Simulation details for calculating the solvation free energy difference (Δ*F*) by thermodynamic integration

Thermodynamic integration (TI)[1] was used to calculate the free energy difference between two given states (A and B) with potential energies U_{A} and U_{B}, respectively. In our case, state A consists of 501 HFO-1234ze(E) and 500 HFO-1234yf molecules, whereas state B consists of 500 HFO-1234ze(E) and 501 HFO-1234yf molecules. Based on Kirkwood′s coupling parameter approach, TI introduces an auxiliary system whose potential energy $U(\lambda)$ depends linearly on a coupling parameter $\lambda \in [0,1]$. For $\lambda = 0$, the auxiliary system corresponds to the reference state (system A), whereas for $\lambda = 1$, it becomes the target state (system B).

$$\begin{aligned} U(\lambda) &= (1-\lambda)U_{\mathrm{A}} + \lambda U_{\mathrm{B}} \\ &= U_{A} + \lambda\left(U_{\mathrm{A}} - U_{\mathrm{B}}\right) \end{aligned} \tag{S1}$$

Then, the free energy difference between states A and B can be obtained by

$$\Delta F(A \to B) = \int_0^1 \left\langle \frac{\partial U(\lambda)}{\partial \lambda} \right\rangle_{\lambda} d\lambda = \int_0^1 \left\langle U_{\mathrm{B}} - U_{\mathrm{A}} \right\rangle_{\lambda} d\lambda \tag{S2}$$

The system volume was determined based on the saturated liquid density reported in Table S11. Temperature was maintained at 343 K and 19.5 bar. The Lennard-Jones potential was smoothly switched to zero between 2.5 and 2.4 nm in each direction of the simulation box. Periodic boundary conditions (PBCs) were applied in all three spatial directions to represent bulk-phase behavior. Long-range Coulomb interactions were treated using the Particle Mesh Ewald (PME) method with a real-space cutoff of 2.4 nm and a fractional error tolerance of 10^{-5}. The system was first equilibrated for 1 ns at coupling parameter $\lambda = 0$. Subsequently, 20 independent configurations were selected at 1 ns intervals. Starting from each configuration, separate simulations were performed in which λ was increased individually to 1.0 in increments of 0.02. At each λ value, a 100 ps production simulation was performed. The free energy change was then evaluated according to Eq. (S2).

Reference

(1) Frenkel, D.; Smit, B. *Understanding Molecular Simulation: From Algorithms to Applications*, 2nd ed.; Computational Science Series; Academic Press: San Diego, CA, 2002.
